# Carotenoid productivity in human intestinal bacteria *Eubacterium limosum* and *Leuconostoc mesenteroides* with functional analysis of their carotenoid biosynthesis genes

**DOI:** 10.1016/j.engmic.2024.100147

**Published:** 2024-03-28

**Authors:** Wataru Matsumoto, Miho Takemura, Haruka Nanaura, Yuta Ami, Takashi Maoka, Kazutoshi Shindo, Shin Kurihara, Norihiko Misawa

**Affiliations:** a Research Institute for Bioresources and Biotechnology, Ishikawa Prefectural University, 1-308, Suematsu, Nonoich-shi 921-8836, Japan; b Department of Science and Technology on Food Safety, Kinki University, 930 Nishimitani, Kinokawa, Wakayama 649-6493, Japan; c Research Institute for Production Development, Shimogamo-morimotocho, Sakyo-ku, Kyoto 606-0805, Japan; d Department of Food and Nutrition, Japan Women's University, Mejirodai, Bunkyo-ku, Tokyo 112-8681, Japan

**Keywords:** C_30_ carotenoid biosynthesis, Obligate anaerobe, Human gut bacterium, *Eubacterium limosum*, *Leuconostoc mesenteroides*, *Lactiplantibacillus plantarum*

## Abstract

The human intestinal microbiota that comprise over 1,000 species thrive in dark and anaerobic environments. They are recognized for the production of diverse low-molecular-weight metabolites crucial to human health and diseases. Carotenoids, low-molecular-weight pigments known for their antioxidative activity, are delivered to humans through oral intake. However, it remains unclear whether human intestinal bacteria biosynthesize carotenoids as part of the *in-situ* microbiota. In this study, we investigated carotenoid synthesis genes in various human gut and probiotic bacteria. As a result, novel candidates, the *crtM* and *crtN* genes, were identified in the carbon monoxide-utilizing gut anaerobe *Eubacterium limosum* and the lactic acid bacterium *Leuconostoc mesenteroides* subsp. *mesenteroides*. These gene candidates were isolated, introduced into *Escherichia coli*, which synthesized a carotenoid substrate, and cultured aerobically. Structural analysis of the resulting carotenoids revealed that the *crtM* and *crtN* gene candidates of *E. limosum* and L. *mesenteroides* mediate the production of 4,4′-diaponeurosporene through 15-*cis*-4,4′-diapophytoene. Evaluation of the *crtE*-homologous genes in these bacteria indicated their non-functionality for C_40_-carotenoid production. *E. limosum* and L. *mesenteroides*, along with the known carotenogenic lactic acid bacterium *Lactiplantibacillus plantarum*, were observed to produce no carotenoids under strictly anaerobic conditions. The two lactic acid bacteria synthesized detectable levels of 4,4′-diaponeurosporene under semi-aerobic conditions. The findings highlight that the obligate anaerobe *E. limosum* retains aerobically functional C_30_-carotenoid biosynthesis genes, potentially with no immediate self-utility, suggesting an evolutionary direction in carotenoid biosynthesis. (229 words)

## Highlights

• Discovery of carotenoid biosynthesis genes in obligatory anaerobic human gut bacteria.
• Functional identification of C_30_ carotenogenic genes from *eubacterium limosum* and *leuconostoc mesenteroides*.
• Probability that carotenoids are not biosynthesized in human gut microbiota.
• Indication of a direction of evolution in the carotenoid biosynthesis.

## Graphical abstract

**Figure:**
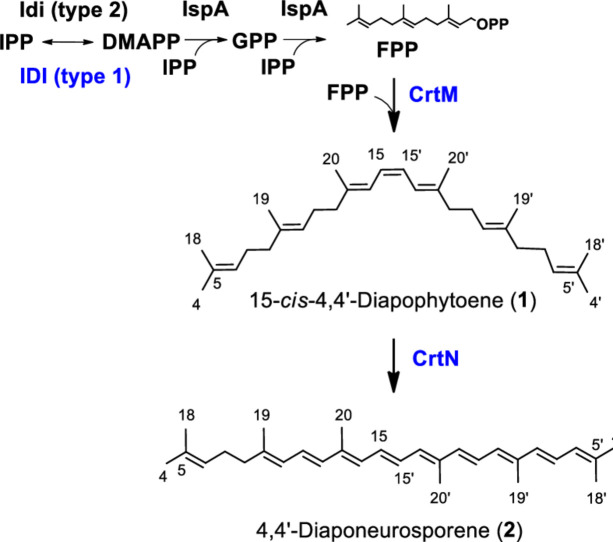

## 1 Introduction

The human intestinal microbiota that inhabit dark and anaerobic environments encompass over 1000 bacterial species. Majority of them belong to the phyla Firmicutes, Bacteroidetes, Actinobacteria, or Proteobacteria [1,2]. This microbial community is recognized for producing diverse low-molecular-weight metabolites, including short-chain fatty acids and polyamines. These metabolites play pivotal roles in host health and diseases, influencing conditions such as inflammatory bowel diseases, obesity, diabetes, and cancers, particularly colon cancer [3], [4], [5], [6], [7], [8], [9].

Carotenoids, characterized by their yellow, orange, or red hues, are low-molecular-weight compounds (isoprenoids) with extensive conjugated double bonds. They are synthesized by certain fungi, archaea, bacteria, as well as all plants and algae [10], [11], [12], [13], [14], [15]. Carotenogenic bacteria, capable of biosynthesizing carotenoids, span various phyla, including Firmicutes (e.g., *Staphylococcus aureus, Planococcus maritimus*, and *Lactiplantibacillus plantarum*), Bacteroidetes (*Flavobacterium glycines* and *Jejuia pallidilutea*), Actinobacteria (*Streptomyces griseus, Arthrobacter glacialis*, and *Micrococcus luteus*), Proteobacteria (*Paracoccus carotinifaciens, Rhodobacter capsulatus*, and *Pantoea ananatis*), Deinococcus-Thermus (*Deinococcus radiodurans* and *Thermus thermophilus*), Verrucomicrobia (*Rubritalea squalenifaciens*), and Cyanobacteria (all species) [11,[15], [16], [17], [18]]. Unlike plants and algae, humans, like other animals, cannot synthesize carotenoids and must acquire them from dietary sources such as vegetables and fruits. While gut microbiota receive and metabolize dietary carbon 40 (C_40_) carotenoids, evidence regarding how human microbes contribute to carotenoid bioavailability remains scarce, despite the identification of some transporters facilitating carotenoid uptake in intestinal cells [19].

Carotenoid pigments function as antioxidants, combating singlet oxygen and lipid peroxidation, thereby imparting various health benefits to humans. These benefits include preventive measures against fatty liver disease and diabetic kidney disease, as well as exhibiting anticancer and anti-inflammatory effects [12,15,[20], [21], [22], [23], [24]].

The synthesis of carotenoids by human intestinal bacteria remains uncertain [9]. Considering that these bacteria inhabit dark, anaerobic environments, they might not have had the opportunity to evolve the capacity for carotenoid production. This speculation aligns with the primary role of carotenoids, which is to shield organisms from photooxidative damage induced by light [20]. Conversely, if human intestinal microbiota were capable of carotenoid production, they could serve as a source for supplying these compounds to humans.

Certain non-photosynthetic bacteria within the Firmicutes phylum, including lactic acid bacteria, can biosynthesize acyclic carotenoids with a carbon 30 (C_30_) basic structure instead of typical C_40_-carotenoids [11,15,25,26]. Recent suggestions propose that C_30_ carotenoids originated from a squalene synthetic route [27]. In this study, we investigated the presence of carotenogenic bacteria among various gut and probiotic bacteria. Subsequently, gene candidates for C_30_ carotenoid biosynthesis were identified in the obligatory anaerobic human gut bacterium *Eubacterium limosum* and the lactic acid bacterium *Leuconostoc mesenteroides.* We functionally identified these biosynthesis gene candidates and cultured these human intestinal bacteria under strictly anaerobic conditions. Evaluation of carotenoid productivity indicated a likelihood that carotenoids are not biosynthesized in the human gut microbiota. Additionally, we explored the evolutionary direction of carotenoid biosynthesis.

## 2 Materials and methods

### 2.1 Intestinal bacterial strains and culture conditions

For this study, we selected 32 gut bacterial strains culturable in Gifu Anaerobic Medium (GAM; Nissui Pharmaceutical, Tokyo, Japan) [28], chosen from the top 56 dominant species listed in the “human gut microbial gene catalogue” [1]. Additionally, we selected 11 other prevalent gut-bacterial species in humans, as outlined in Table 1. We also examined eight lactic acid bacteria and nine Bifidobacteria as human probiotic bacteria (Table 1).

**Table 1:** Bacterial strains used in this study, which are the inhabitants of the human intestine, and carotenoid biosynthesis gene homologues found in genome data bases .

Species	Strain	Genome sequence	Rank*	Found carotenogenic gene (Accession No.)
Human gut bacterium				
*Bacteroides uniformis*	JCM 5828		1	
*Parabacteroides merdae*	JCM 9497		3	
*Dorea longicatena*	DSM 13814		4	
*Bacteroides caccae*	JCM 9498		6	
*Bacteroides thetaiotaomicron*	JCM 5897	NC_004663.1	8	
*Ruminococcus torques*	ATCC 27756		10	
*Ruminococcus lactaris*	ATCC 29176		14	
*Collinsella aerofaciens*	JCM 7790		15	
*Dorea formicigenerans*	ATCC 27755		16	
*Bacteroides vulgatus*	JCM 5826	NC_009614.1	17	*crtN*-like (ABR38724.1)
*Roseburia intestinalis*	DSM 14610	NZ_LR027880.1	18	
*Eubacterium siraeum*	ATCC 29066		20	
*Parabacteroides distasonis*	JCM 5825	NC_009615.1	21	*crtN*-like (ABR42459.1)
*Bacteroides ovatus*	JCM 5824	NZ_CP012938.1	23	*crtN*-like (ALJ48660.1)
*Bacteroides xylanisolvens*	JCM 15633		27	
*Coprococcus comes*	ATCC 27758		28	
*Eubacterium ventriosum*	ATCC 27560		31	
*Bacteroides dorei*	JCM 13471	NZ_LR699004.1	32	*crtN*-like (AII67407.1)
*Ruminococcus obeum*	DSM 25238		33	
*Pseudoflavonifractor capillosus*	ATCC 29799		35	
*Bacteroides stercoris*	JCM 9496		39	
*Bacteroides finegoldii*	JCM 13345		44	
*Parabacteroides johnsonii*	JCM 13406		45	
*Clostridium nexile*	ATCC 27757		47	
*Anaerotruncus colihominis*	JCM 15631		49	
*Ruminococcus gnavus*	ATCC 29149	NZ_CP027002.1	50	
*Bacteroides intestinalis*	JCM 13265		51	
*Bacteroides fragilis*	JCM 11019	NC_006347.1	52	
*Clostridium asparagiforme*	DSM 15981		53	
*Enterococcus faecalis* V583	ATCC 700,802	NC_004668.1	54	
*Clostridium scindens*	JCM 6567	NZ_CP036170.1	55	
*Blautia hansenii*	JCM 14655	NZ_CP022413.2	56	
*Fusobacterium nucleatum* subsp. *nucleatum*	JCM 8532^T^	NC_003454.1		
*Clostridium ramosum*	JCM 1298			
*Clostridium indolis*	JCM 1380			
*Clostridium bolteae*	JCM 12243	NZ_CP022464.2		
*Clostridium perfringens*	JCM 1290	NC_008261.1		*crtN*-like (ABG84740.1)
*Clostridium difficile*	JCM 1296^T^	NC_009089.1		
*Eubacterium cylindroides*	JCM10261^T^			
*Eubacterium limosum*	ATCC 8486	NZ_CP019962.1		*crtM* (ARD66429.1), *crtN* (ARD66430.1), *crtE* (ARD65667.1)
*Fusobacterium varium*	ATCC 27725	NZ_CP028103.1		
*Citrobacter youngae*	ATCC 29220			
*Ruminococcus productus*	JCM 1471	NZ_CP039126.1		
Lactic acid bacterium⁎⁎				
*Lactobacillus* (*Lacticaseibacillus*) *casei* subsp. *casei*	JCM 1134	NZ_AP012544.1		
*Lactobacillus* (*Lacticaseibacillus*) *casei* subsp*. rhamnosus*	ATCC 7469			
*Lactobacillus johnsonii*	JCM 8794			
*Lactobacillus* (*Lactiplantibacillus*) *plantarum*	JCM 1149	NC_004567.2		*crtM* (AJO75313.1), *crtN* (AJO75312.1), *crtE* (AJO73620.1)
*Lactobacillus* (*Limosilactobacillus*) *reuteri*	JCM 1112	NC_009513.1		
*Lactococcus lactis*	JCM 1158	NC_022369.1		
*Lactobacillus gasseri*	JCM 1130	NC_008530.1		
*Leuconostoc mesenteroides* subsp. *mesenteroides*	JCM 6124	NC_008531.1		*crtM* (ABJ62147.1), *crtN* (ABJ62146.1), *crtE* (ABJ61354.1)
Bifidobacterium				
*Bifidobacterium adolescentis*	JCM 1275^T^	NC_008618.1		
*Bifidobacterium animalis* subsp. *lactis*	JCM 10602^T^	NC_012815.1		
*Bifidobacterium bifidum*	JCM 1254	NC_014638.1		
*Bifidobacterium breve*	JCM 1192^T^	NZ_CP006712.1		
*Bifidobacterium catenulatum*	JCM 1194^T^	NZ_AP012325.1		
*Bifidobacterium longum*	JCM 1217^T^	NC_004307.2		
*Bifidobacterium pseudocatenulatum*	JCM 1200^T^	NZ_CP025199.1		
*Bifidobacterium pseudolongum*	JCM 1205^T^	NZ_CP022544.1		
*Bifidobacterium infantis*	ATCC 15697^T^	NC_004307.2		

⁎ Rank shows the order of bacterial cell abundance in human gut [1].

⁎⁎ Genera names according to new reclassification of lactic acid bacteria are shown in parentheses [23].

These bacteria were routinely cultured under strictly anaerobic conditions, maintaining the entire process within an anaerobic chamber (INVIVO_2_ 400, Baker Ruskinn, Sanford, ME, USA) [28] as outlined below:

1. Pre-preculture (Large Scale Cultivation, 1 L):

• Bacterial glycerol stocks (small amounts) were individually inoculated into the GB [GAM and 10 % (v/v) horse blood] liquid medium inside the anaerobic chamber.
• Cultured without agitations (statically) at 37 °C for 24 h.
• Pre-precultures were carried out only in large-scale cultivation.

2. Preculture:

• 1 % of the pre-precultures or the glycerol stocks were transferred into the GAM liquid medium and statically cultured at 37 °C for 24 or 48 h.
• 1 % of the bacterial precultures were individually transferred into the GAM liquid medium and statically incubated at 37 °C for 24 h.
• Throughout this cultivation, the materials were continuously kept in the anaerobic chamber (referred to as “the (strictly) anaerobic condition”).

3. Semi-anaerobic Condition:

• As needed, 1 % of the bacterial precultures were inoculated into the GAM liquid medium in the anaerobic chamber and statically incubated at 37 °C for 24 or 48 h outside the anaerobic chamber (referred to as "the semi-anaerobic condition").

4. Semi-aerobic Condition:

• Alternatively, 1 % of the bacterial precultures were inoculated into the GAM liquid medium outside the anaerobic chamber and statically incubated at 37 °C for 24 or 48 h outside the anaerobic chamber (referred to as "the semi-aerobic condition").

For large-scale cultivation (1 L), 16S rDNA of the cultured bacterial cells was amplified by PCR using primer 7F (5′-AGAGTTTGATYMTGGCTCAG-3′) and primer 1510R (5′-ACGGYTACCTTGTTACGACTT-3′). The amplified DNA fragments were sequenced, followed by alignment analysis, to confirm the absence of contamination.

Regarding DNA preparation and carotenoid extraction, *Lactobacillus plantarum* (recently reclassified to L. *plantarum* [29]), L. *mesenteroides* subsp. *mesenteroides, Bacteroides vulgatus, Parabacteroides distasonis, Bacteroides ovatus, Bacteroides dorei*, and *Clostridium perfringens* were cultured under semi-aerobic conditions, while *E. limosum* was grown under strictly anaerobic conditions.

### 2.2 Escherichia coli strains and culture conditions

*E. coli* DH5α and *E. coli* JM101 (DE3) [30] were employed for plasmid analysis and carotenoid biosynthesis, respectively. The culture conditions were as follows: Transformed *E. coli* DH5α cells were cultured in 2YT medium (1.6 % Bactotryptone, 1 % yeast extract, 0.5 % NaCl), supplemented with tetracycline (Tc; 10 mg/L) [or ampicillin (Ap; 40 mg/L) or chloramphenicol (Cm; 30 mg/L)], at 37 °C for 16 h. Recombinant *E. coli* JM101 (DE3) was grown in 2YT medium containing Tc or Cm, along with Ap as needed, at 37 °C for 16 h. Subsequently, this culture was inoculated into the same medium with 0.05 mM IPTG (isopropyl thiogalactoside) to induce the *tac* and *lac* promoters, and cultured at 20 °C for 48 h. All *E. coli* cells were aerobically cultured with agitation.

### 2.3 Sequence analysis and cloning of carotenoid biosynthesis gene candidates

To identify nucleotide sequences homologous to known bacterial carotenoid biosynthesis genes, BLAST searches were conducted in individual genome databases of intestinal bacteria (http://blast.ncbi.nlm.nih.gov/Blast.cgi). The searched genes included *crtM, crtN, crtE, crtB, crtI, crtY*, and *crtZ*. Nucleotide sequences of the obtained carotenogenic gene candidates were analyzed using DNASIS DNA analysis software (Hitachi Solutions, Tokyo, Japan).

Genomic DNAs of intestinal bacteria were extracted using a phenol/chloroform extraction method, as reported previously [31]. Primer sequences were designed based on gene sequences homologous to carotenoid biosynthesis genes found in the genomes (*crtM, crtN*, or *crtE*; Table 1). Carotenogenic gene candidates were amplified by PCR, using PrimeSTARⓇ GXL Polymerase (Takara Bio, Ohtsu, Japan). The PCR products were inserted into *E. coli* vectors such as pBC KS (Agilent, CA, USA) and introduced into *E. coli* DH5α. Plasmid DNAs were extracted from the transformed *E. coli* cells and subjected to DNA sequencing with ABI3130 × 1 (Applied Biosystems, CA, USA).

### 2.4 Phylogenetic tree construction

Alignment and phylogenetic reconstructions were conducted using the “build” function of ETE3 3.1.2 [32] available on GenomeNet (https://www.genome.jp/tools/ete/). The Maximum Likelihood (ML) tree was inferred using IQ-TREE 1.5.5, with ModelFinder for tree reconstruction [33]. Tree branches were tested using SH-like aLRT with 1000 replicates.

### 2.5 Plasmid construction

**Fig. 1:**
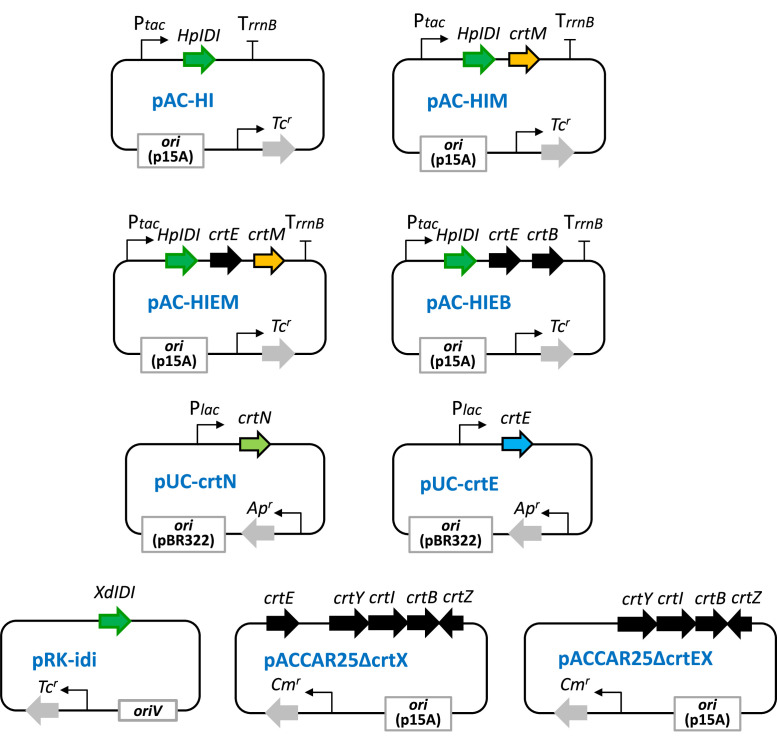
Structure of plasmids used in this study.

The detailed construction method for the plasmid named pAC—HI is described, containing the *IDI* (isopentenyl diphosphate isomerase, type 1) gene from green alga *Haematococcus pluvialis* [34]. This gene was placed within the *tac* promoter (P*tac*) and the *rrnB* terminator (T*rrnB*) using the pACYC184 vector [35]. The *tac* promoter fragment was PCR-amplified using primer sets, PtacF: 5′-CGAATTCAGCTGTTGACAATTAATC-3′ and PtacR: 5′-AGGTACCGCTCGAGTGTTTCCTGTGTGAAATTG-3′. The *rrnB* terminator was PCR-amplified using primers, TrrnBF: 5′-CGGTACCCTGTTTTGGCGGATGAGAG-3′ and TrrnBR: 5′-CCCATGGCTGCTTTCCTGATGCAAAAAC-3′. The P*tac* and T*rrnB* fragments were inserted into the *Eco*RI and *Nco*I sites of pACYC184, disrupting the chloramphenicol-resistant gene. The *H. pluvialis IDI* gene was PCR-amplified using primers, HpIDIF: 5′-ACTCGAGGAGGCAGCTATGCTTCGTTCGTTGCTC-3′ and HpIDIR: AGGTACCCAGATCTTACGCTTCGTTGATGTG-3′. This fragment was inserted into the *Xho*I and *Kpn*I sites between P*tac* and T*rrnB*, creating plasmid pAC—HI (Fig. 1) (accession, LC635748), used as the base plasmid for constructing plasmids pAC—HI series [36].

Each isolated *crtM* gene candidate was inserted between the *Bgl*II and *Kpn*I sites of pAC—HI, resulting in plasmids pAC—HIM series (Fig. 1). Each isolated *crtN* gene candidate was inserted between the *Eco*RI and *Hin*dIII or *Kpn*I sites of pUC18 vector (Takara Bio, Ohtsu, Japan), creating plasmids pUC-crtN series. Each isolated *crtE* gene candidate was cloned between the *Bam*HI and *Kpn*I sites of pUC19 (Takara Bio, Ohtsu, Japan), creating pUC-crtE series. Plasmids pAC—HIEM series, where the coding region of the *crtE* gene derived from *P. ananatis* was introduced into pAC—HIM series, were also constructed. Plasmid pAC—HIEB was constructed to express the *P. ananatis crtE* and *crtB* genes as described [36] (Fig. 1). Plasmid pACCAR25ΔcrtEX expressing the *crtB, crtI, crtY*, and *crtZ* genes derived from *P. ananatis* was constructed by disrupting the *crtE* gene from pACCAR25ΔcrtX (accession, LC642800) [37], and pRK-idi (accession, LC645088) contained the *IDI* gene from fungus *Xanthophyllomyces dendrorhous* [38].

### 2.6 Construction of recombinant E. coli for carotenoid biosynthesis

The pAC—HIM and pAC—HIEM series were introduced into *E. coli* JM101 (DE3)*.* The pUC-crtN series were introduced into *E. coli* JM101 (DE3) containing plasmid pAC—HIM (from the L. *plantarum crtM*) or pAC—HIEB. The pUC-crtE series were introduced into *E. coli* JM101 (DE3) possessing two plasmids pACCAR25ΔcrtEX and pRK-idi. Empty vectors were introduced into *E. coli* JM101 (DE3) as a negative control.

### 2.7 Carotenoid extraction and HPLC-PDA analysis

The bacterial cultures, including recombinant *E. coli* culture, underwent centrifugation, and methanol was added to the cell pellets. Tris–HCl (pH 7.5), NaCl, and chloroform were added to the solution, mixed for 5 min, and centrifuged. The upper phase was collected and dried by centrifugal evaporation. The pellet was suspended with ethyl acetate (EtOAc) and applied to HPLC using a Waters Alliance 2695–2996 system (photodiode array detector; PDA) (Waters, Milford, MA, USA). The HPLC analysis used a TSKgel ODS-80 s column (4.6 × 150 nm, 5 μm; Tosoh, Tokyo, Japan) with the following method: Samples were eluted at a flow rate of 1.0 mL/min at 25 °C with two solvents – solvent A [water (H_2_O)-methanol (MeOH), 5:95, v/v] and solvent B (tetrahydrofuran-MeOH, 30:70, v/v). Solvent A was perfused for 5 min, followed by a linear gradient from solvent A to solvent B for 5 min, and then solvent B only for 8 min.

Additionally, carotenoids were extracted and analyzed from intestinal bacterial cells using the routine method of Maoka's lab [36]. The HPLC/MS analysis of carotenoids was performed using a Waters Xevo G2S Q TOF mass spectrometer (Waters Corporation, Milford, CT, USA) equipped with an Acquity UPLC (HPLC) system. UV–VIS absorption spectra were recorded from 200 to 600 nm using a photodiode-array detector (PDA). An Acquity 1.7 μm BEH UPLC C18 (2.1 id X 100 mm) column (Waters Corporation, Milford, CT, USA) was used for the HPLC system, developed by acetonitrile (MeCN):H_2_O (85:15) - MeCN:MeOH (65:35) (linear gradient 0 to 15 min) as a mobile phase, at a flow rate of 0.4 mL/min.

Carotenoids were identified by comparing retention times and absorption spectra with those of authentic samples purified from recombinant *E. coli* and confirmed by UV–visible, MS/MS, and NMR analyses [36].

### 2.8 Purification of carotenoids

#### 2.8.1 Isolation of 15-*cis*-4,4′-diapophytoene (**1**)

The transformed *E. coli* cells carrying pAC—HIM (from the L. *plantarum crtM*) from a 2 L culture were collected by centrifugation at 13,000 × *g* for 5 min. After removing the supernatant, the produced compounds were extracted with 50 mL acetone-MeOH (7:2) through sonication (× 3). The combined extract (150 mL) was concentrated to a small volume in vacuo, and partitioned between *n*-hexane/90 % MeOH (each 100 mL). The *n*-hexane layer was evaporated to dryness (36.2 mg) and subjected to silica gel chromatography (15 × 150 mm) (CHROMATOREX FL60D, Fuji Silysia Chemical Ltd, Aichi, Japan) using *n*-hexane, and fractionated by 10 mL. The fractions 6–9 containing a UV compound (Rf 0.8 by silica gel (silica gel 60) TLC using developing solvent *n*-hexane-EtOAc (10:1)) were collected and concentrated to afford pure 15-*cis*-4,4′-diapophytoene (1, 9.8 mg).

#### 2.8.2 Isolation of 4,4′-diaponeurosporene (**2**)

The transformed *E. coli* cells carrying pAC—HIM (from the L. *plantarum crtM*) and pUC-crtN (from the L. *mesenteroides* subsp. *mesenteroides crtN*) from a 3 L culture were collected, extracted, and partitioned as described in the isolation of 15-*cis*-diapo-4,4′-phytoene. The *n*-hexane layer was evaporated to dryness (13.6 mg) and subjected to silica gel chromatography (15 × 150 mm) using *n*-hexane, and fractionated by 10 mL. The fractions 25–32 containing a yellow compound (Rf 0.7 by silica gel TLC using developing solvent *n*-hexane-EtOAc (10:1)) were collected and concentrated to afford pure 4,4′-diaponeurosporene (**2**, 2.5 mg).

### 2.9 NMR analysis of carotenoids and their NMR spectroscopic data

NMR spectra were acquired using an AVANCE400 instrument (Bruker BioSpin, Karlsruhe, Germany) in CDCl_3_, with the residual solvent peak serving as an internal standard (δ_C_ 77.0, δ_H_ 7.26 ppm).

### 2.10 ^1^H and ^13^C NMR data of 15-*cis*-4,4′-diapophytoene (**1**)

^1^H NMR (CDCl_3_) δ: 1.60 (6H, s, H-18 and H-18′), 1.61 (6H, s, H-19 and H-19′), 1.68 (6H, s, H-4 and H-4′), 1.77 (6H, s, H-20 and H-20′), 1.98 (4H, m, H-8 and H-8′), 2.05 (4H, m, H-7 and H-7′), 2.15 (8H, H-11, H-11′, H-12, and H-12′), 5.09 (2H, m, H-6 and H-6′), 5.11 (2H, m, H-10 and H-10′), 6.10 (2H, m, H-15 and H-15′), 6.30 (2H, m, H-14 and H-14′).

^13^C NMR (CDCl_3_) δ: 16.0 (C-19 and C-19′), 16.5 (C-20 and C-20′), 17.7 (C-18 and C-18′), 25.7 (C-4 and C-4′), 26.7 (C-11 and C-11′), 26.8 (C-7 and C-7′), 39.7 (C-8 and C-8′), 40.5 (C-12 and C-12′), 120.2 (C-14 and C-14′), 123.3 (C-15 and C-15′), 124.0 (C-10 and C-10′), 124.4 (C-6 and C-6′), 131.3 (C-5 and C-5′), 135.3 (C-9 and C-9′), 139.5 (C-13 and C-13′).

### 2.11 ^1^H and ^13^C NMR data of 4,4′-diaponeurosporene (**2**)

^1^H NMR (CDCl_3_) δ: 1.61 (3H, s, H-18), 1.69 (3H, s, H-4), 1.82 (9H, s, H-19, H-4′, and H-18′), 1.95 (3H, s, H-19′), 1.96 (6H, s, H-20 and H-20′), 2.11 (4H, H-6 and H-7), 5.10 (1H, m, H-6), 5.93 (1H, d, *J* = 9.3 Hz, H-6′), 5.96 (1H, d, *J* = 9.9 Hz, H-10), 6.19 (2H, H-14 and H-14′), 6.22 (1H, d, *J* = 15.1 Hz, H-8′), 6.24 (1H, d, *J* = 14.8 Hz, H-12), 6.25 (1H, d, *J* = 11.8 Hz, H-10′), 6.34 (1H, d, *J* = 14.8 Hz, H-12′), 6.47 (1H, dd, *J* = 9.3, 15.1 Hz, H-7′), 6.49 (1H, dd, *J* = 9.9, 14.8 Hz, H-11), 6.60 (2H, H-15 and H-15′), 6.61 (1H, dd, *J* = 11.8, 14.8 Hz, H-11′).

^13^C NMR (CDCl_3_) δ: 12.8 (C-20 and C-20′), 12.9 (C-19′), 17.0 (C-19), 17.7 (C-18), 18.6 (C-18′), 26.3 (C-4), 26.3 (C-4′), 26.7 (C-7), 40.2 (C-8), 123.9 (C-6), 124.7 (C-7′), 124.9 (C-11′), 125.0 (C-11), 125.7 (C-10), 126.1 (C-6′), 129.5 (C-15′), 130.1 (C-15), 131.5 (C-14), 131.5 (C-14′), 131.7 (C-5), 132.6 (C-10′), 135.0 (C-8′), 135.3 (C-12), 135.8 (C-5′), 136.0 (C-13), 136.1 (C-13′), 136.5 (C-9′), 137.4 (C-12′), 139.7 (C-9).

## 3 Results

### 3.1 Color screening of intestinal bacterial strains

In this study, we carefully selected 43 gut bacteria, eight lactic acid bacteria, and nine Bifidobacteria, all considered inhabitants of the human intestine (Table 1). The question of whether human intestinal bacteria inherently produce carotenoids has been lingering. To investigate this, we assessed the coloration of the selected bacteria in pure cultivations, specifically looking for shades of yellow, orange, or red (pink). Colony pigmentation often serves as an initial indicator of microbial carotenoid production [17]. These bacteria were statically cultured with 0.5 mL in 96-well plates at 37 °C under strictly anaerobic conditions. After growth and centrifugation, the bacterial pellets, including L. *plantarum*, a known carrier of carotenogenic genes [25,39], exhibited no apparent coloration (Fig. S1).

### 3.2 Search of carotenoid biosynthesis genes

Our subsequent efforts focused on identifying carotenoid biosynthesis genes within the selected bacterial strains. Utilizing the NCBI genome database (https://www.ncbi.nlm.nih.gov/genome/), we accessed 33 available genome sequences of the selected intestinal bacteria Table 1. Homology searches unveiled that three bacteria retained nucleotide sequences homologous to known bacterial carotenoid biosynthesis genes, specifically *crtM* (*crtB*), *crtN* (*crtI*), and *crtE*. Notably, sequences homologous to other bacterial carotenogenic genes, such as *crtY* and *crtZ*, were not detected in these genome sequences. Additionally, five bacteria were found to possess only a *crtN*-like gene.

L. *plantarum* (belonging to the Lacobacillaceae family, class Bacilli, phylum Firmicutes) harboring the *crtM* and *crtN* genes has been reported to have the capability to produce 4,4′-diaponeurosporene [23,37]. In our current study, we made a discovery of *crtM* and *crtN* homologs (orthologs) in the gut bacterium *E. limosum* (Eubacteriaceae family, class Clostridia, phylum Firmicutes), in addition to the lactic acid bacterium L. *mesenteroides* subsp. *mesenteroides* (Lacobacillaceae family; [29]), alongside L. *plantarum*, as detailed in Table 1.

The CrtM proteins deduced from the *crtM* genes of *E. limosum*, L. *mesenteroides*, and L. *plantarum* exhibited amino acid identities of 29.6 %, 25.6 %, and 28.3 % to the CrtM of *S. aureus* (Staphylococcaceae family; class Bacilli; [40]), respectively. Similarly, the CrtN proteins from *E. limosum*, L. *mesenteroides*, and L. *plantarum* displayed moderately high homologies, with amino acid identities of 45.8 %, 40.4 %, and 44.6 % to the *S. aureus* CrtN protein, respectively. Additionally, *crtN*-like genes (ORFs) were identified in the genomes of *B. vulgatus, P. distasonis, B. ovatus, B. dorei*, and *C. perfringens*, although their amino acid sequences exhibited only approximately 16 % homology to the *S. aureus* CrtN.

Nucleotide sequences similar to *crtE* were also detected in *E. limosum*, L. *mesenteroides,* and L. *plantarum*. These respective amino acid sequences retained identities of 31.4 %, 27.4 %, and 30.4 % to the *P. ananatis* CrtE.

### 3.3 Carotenoid productivity of intestinal bacteria retaining carotenoid biosynthesis genes

**Fig. 2:**
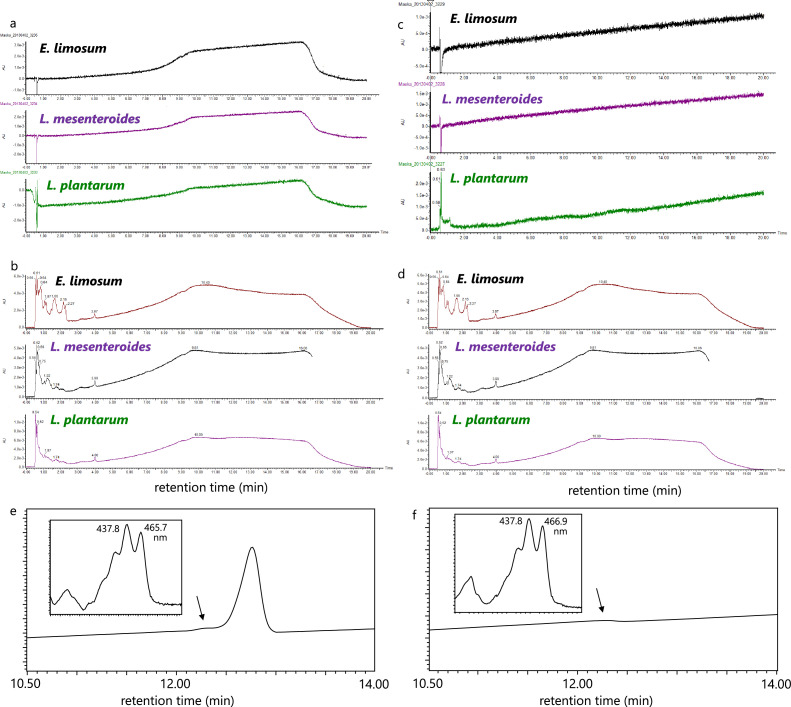
HPLC chromatograms of extracts from *Eubacterium limosum, Leuconostoc mesenteroides*, and *Lactiplantibacillus plantarum*, cultured under strictly anaerobic (**a**, at 450 nm; **b**, at 280 nm) and semi-anaerobic conditions (**c**, at 450 nm; **d**, at 280 nm), and HPLC chromatograms with UV spectra of extracts from L. *mesenteroides* (**e**) and L. *plantarum* (**f**), cultured under semi-aerobic conditions.

The gut bacterium *E. limosum* and lactic acid bacteria, namely, L. *mesenteroides* and L. *plantarum*, although lacking visible carotenoid coloration, may still produce trace amounts of C_30_-carotenoids in their anaerobic environments due to the presence of the C_30_-carotenoid biosynthesis *crtM* and *crtN* genes. To investigate this possibility, we assessed the anaerobic carotenoid production by these human intestinal bacteria. The three bacterial strains were statically cultured for 24 h under both anaerobic and semi-aerobic conditions, employing Erlenmeyer flasks containing 1 L of GAM medium. The fresh weights of the bacterial cells centrifuged from the anaerobic (and semi-anaerobic) cultures of *E. limosum*, L. *mesenteroides*, and L. *plantarum* were 1.57 g (0.50 g), 0.23 g (0.20 g), and 0.47 g (1.48 g), respectively. Each culture was verified not to include contaminated cells through 16S DNA sequence analysis (Table S2). Subsequently, each cell pellet was extracted with solvents such as acetone and analyzed using the routine method of Maoka's lab [36]. The HPLC chromatograms (Fig. 2a–d) revealed that *E. limosum*, L. *mesenteroides*, and L. *plantarum* did not exhibit carotenoid peaks at 450 nm or 280 nm, nor did they display peaks of 4,4′-diaponeurosporene or 4,4′-diapophytoene. Further analysis in the semi-aerobic condition confirmed that L. *mesenteroides* and L. *plantarum* could produce detectable levels of 4,4′-diaponeurosporene (Fig. 2e,f).

### 3.4 Isolation of carotenoid biosynthesis gene candidates

All the *crtM, crtN*, and *crtE* gene candidates, as well as *crtN*-like genes identified from the genome sequences in Table 1, were isolated through PCR using the primer sets (Table S1), as detailed in the Materials and Methods section.

### 3.5 Functional analysis of crtM gene candidates

**Fig. 3:**
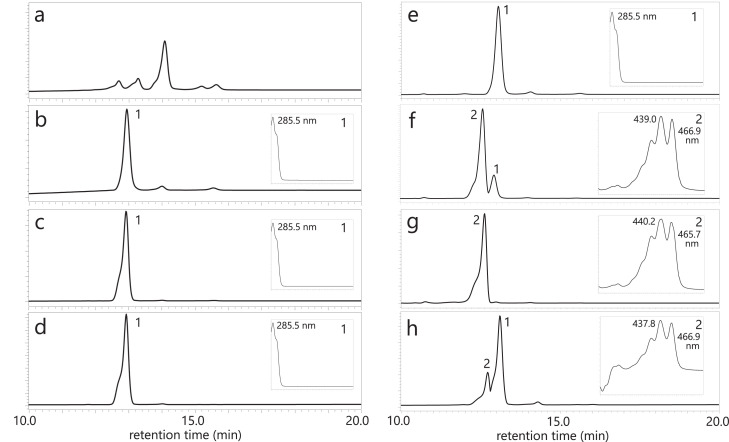
HPLC chromatograms with UV spectra of extracts from *Escherichia coli* cells retaining the control plasmid pAC—HI (no *crtM*) (**a**), plasmid pAC—HIM (*E. limosum crtM*) (**b**), plasmid pAC—HIM (L. *mesenteroides crtM*) (**c**), and plasmid pAC—HIM (L. *plantarum crtM*) (**d**), and HPLC chromatograms with UV-visible spectra of extracts from *E. coli* cells retaining plasmid pAC—HIM (L. *plantarum crtM*) plus the empty vector pUC18 (no *crtN*) (**e**), plus plasmid pUC-crtN (*E. limosum crtN*) (**f**), plus plasmid pUC-crtN (L. *mesenteroides crtN*) (**g**), and plus plasmid pUC-crtN (L. *plantarum crtN*) (**h**). **1**, 4,4′-diapophytoene; **2**, 4,4′-diaponeurosporene.

In the pursuit of functional insights into the *crtM*-homologous genes (orthologues) from *E. limosum* and L. *mesenteroides*, alongside the L. *plantarum crtM*, we conducted functional characterization using *E. coli*. Plasmids of the pAC—HIM series, which incorporated the *Haematococcs pluvialis IDI* (*HpIDI*) gene and each *crtM* gene, were constructed (Fig. 1) and individually introduced into *E. coli*, followed by HPLC-PDA analysis (Fig. 3). In the control scenario where the pAC—HI plasmid was introduced into *E. coli*, no carotenoid was produced (Fig. 3a). Conversely, the analysis of *E. coli* carrying the pAC—HIM plasmids, containing any *crtM* from the three bacteria, revealed peaks corresponding to 4,4′-diapophytoene (Fig. 3b–d). These findings indicated that the *crtM* gene products (CrtM proteins) of *E. limosum* and L. *mesenteroides* exhibited 4,4′-diapophytoene synthase activity, similar to that of L. *plantarum*.

**Fig. 4:**
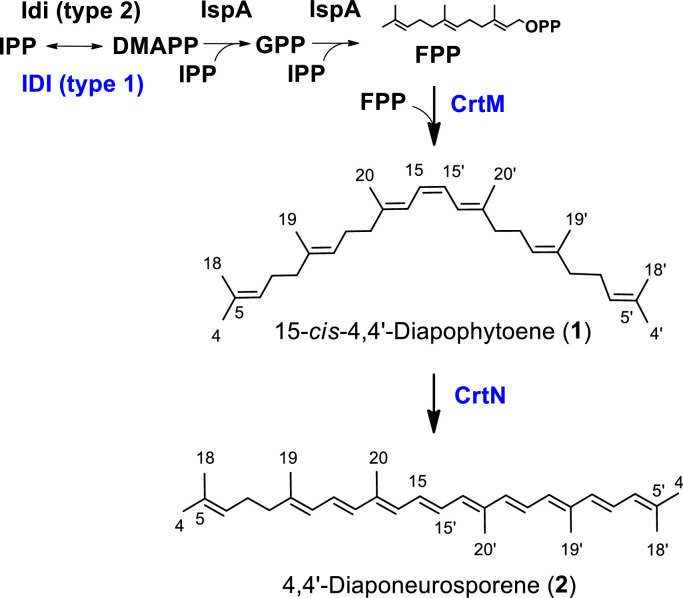
Carotenoid biosynthetic pathway in recombinant *E. coli* for the production of 15-*cis*-4,4′-diapophytoene and 4,4′-diaponeurosporene, with structures determined by NMR. Names of proteins encoded by foreign genes are shown in blue. Idi (type 2), isopentenyl diphosphate (IPP) isomerase type 2; IspA, farnesyl diphosphate (FPP) synthase of *E. coli*; DMAPP, dimethylallyl diphosphate; GPP, geranyl diphosphate.

The transformed *E. coli* carrying pAC—HIM (L. *plantarum crtM*) was further cultured for detailed chemical analysis. The resulting compound was purified from the cells, as described in the Materials and Methods section, and subjected to analysis by ESI-MS (+), ^1^H, and ^13^C NMR. Based on these spectra, the produced chemical compound was identified as 15-*cis*-diapo-4,4′-phytoene (**1**) (Fig. 4) [41]. Since the assignment data for ^1^H and ^13^C NMR have not been completely reported for such heterologous production, we have included them in the Materials and Methods section.

**Fig. 5:**
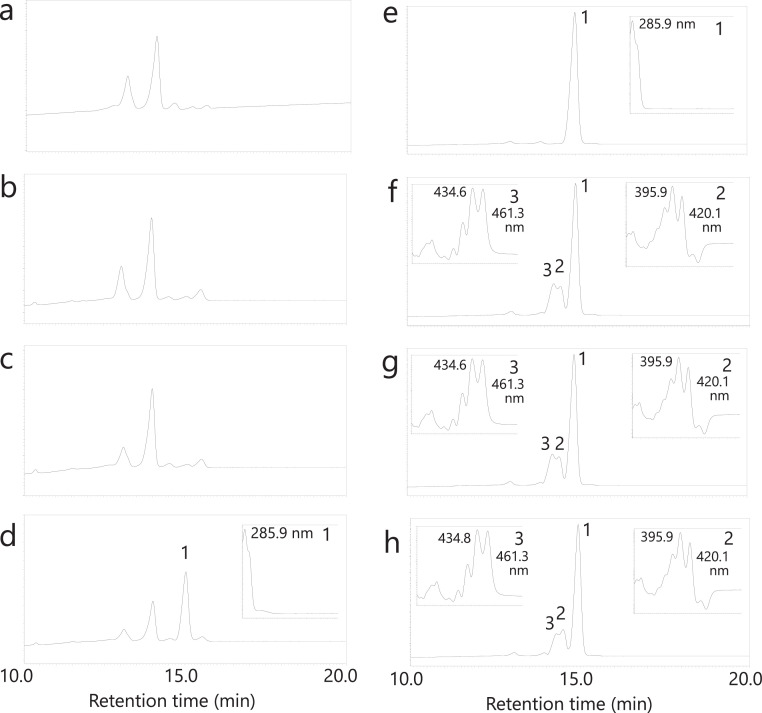
HPLC chromatograms with UV spectra of extracts from *E. coli* cells retaining the control plasmid pAC—HIE (no *crtM*) (**a**), plasmid pAC—HIEM (*E. limosum crtM*) (**b**), plasmid pAC—HIEM (L. *mesenteroides crtM*) (**c**), and plasmid pAC—HIEM (L. *plantarum crtM*) (**d**), and HPLC chromatograms with UV-visible spectra of extracts from *E. coli* cells retaining plasmid pAC—HIEB plus the empty vector pUC18 (no *crtN*) (**e**), plus plasmid pUC-crtN (*E. limosum crtN*) (**f**), plus plasmid pUC-crtN (L. *mesenteroides crtN*) (**g**), and plus plasmid pUC-crtN (L. *plantarum crtN*) (**h**). **1**, phytoene; **2**, ζ-carotene; **3**, neurosporene.

Furthermore, we investigated whether the *crtM* genes from *E. limosum*, L. *mesenteroides*, and L. *plantarum* could convert geranylgeranyl diphosphate (GGPP) using *E. coli* carrying the pAC—HIEM series (Fig. 5a–d). The results revealed that only the L. *plantarum crtM* was capable of mediating the conversion from GGPP to phytoene.

In summary, it was elucidated that the *crtM* genes (candidates) of *E. limosum* and L. *mesenteroides* encode 15-*cis*-4,4′-diapophytoene synthase (Fig. 4), while the L. *plantarum crtM* gene encodes 15-*cis*-4,4′-diapophytoene/phytoene synthase.

### 3.6 Functional analysis of crtN gene candidates

To assess the functions of individual crtN-homologous genes (orthologues), we constructed the pUC-crtN plasmid series (Fig. 1) and independently introduced them into *E. coli* containing pAC—HIM (L. *plantarum crtM*), followed by HPLC-PDA analysis (Fig. 3). Introduction of the empty pUC18 vector into 15-*cis*-4,4′-diapophytoene-producing *E. coli* did not yield any new carotenoid peak (Fig. 3e). However, when the pUC-crtN series, encompassing *E. limosum crtN* (Fig. 3f), L. *mesenteroides crtN* (Fig. 3g), and L. *plantarum crtN* (Fig. 3h), were individually introduced into the 15-*cis*-4,4′-diapophytoene-producing *E. coli*, peaks corresponding to 4,4′-diaponeurosporene were detected in all transformants. These results indicate that the *crtN* gene products (CrtN proteins) of *E. limosum* and L. *mesenteroides* exhibit 4,4′-diapophytoene desaturase activity, akin to L. *plantarum*. Notably, the activity of CrtN enzymes from L. *mesenteroides* and *E. limosum* surpassed that of L. *plantarum* CrtN in *E. coli*.

Conversely, negative results were obtained for the *crtN* homologues derived from *B. dorei* (Fig. S2b), *B. ovatus* (Fig. S2c), *B. vulgatus* (Fig. S2d), *C. perfringens* (Fig. S2e), and *P. distasonis* (Fig. S2f).

For further detailed chemical analysis, we cultured the recombinant *E. coli* cells retaining the plasmids pAC—HIM (L. *plantarum crtM*) and pUC-crtN (L. *mesenteroides crtN*) as a representative, and purified the carotenoid through the aforementioned process. ESI-MS (+), ^1^H, and ^13^C NMR spectral analyses confirmed the compound as (all *trans*)−4,4′-diaponeurosporene (**2**) (Fig. 4) [42]. Since the ^1^H and ^13^C NMR assignment data of **2** have not been reported for such heterologous production, we have included them in the Materials and Methods section. Thus, the *crtN* genes (candidates) of *E. limosum*, L. *mesenteroides*, and L. *plantarum* code for 15-*cis*-4,4′-diapophytoene desaturase was confirmed (Fig. 4).

We further investigated whether the *crtN* genes from *E. limosum*, L. *mesenteroides*, and L. *plantarum* could convert phytoene or not, utilizing *E. coli* carrying the phytoene-synthesizing plasmid pAC—HIEB (Fig. 5e–h). Therefore, all three *crtN* genes were found to mediate inefficient conversion of phytoene to neurosporene via ζ-carotene.

### 3.7 Functional analysis of crtE gene candidates

**Fig. 6:**
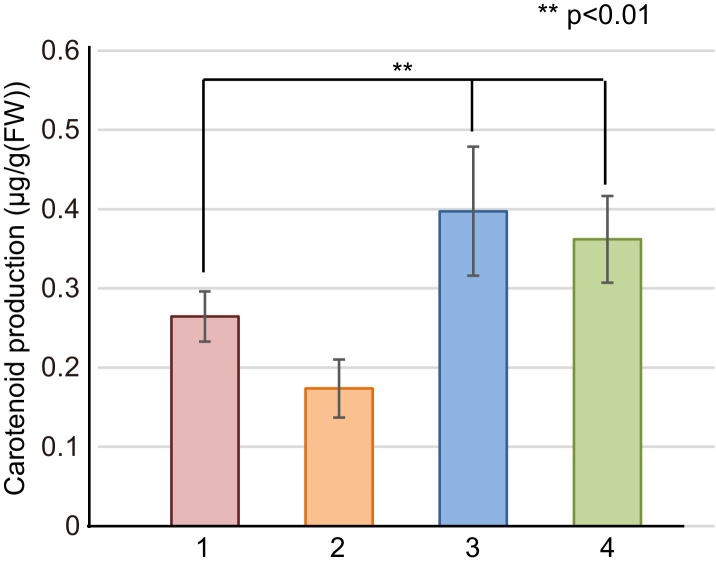
Carotenoid production by *E. coli* cells retaining plasmid pACCAR25ΔcrtEX plus the empty vector pUC19 (1), plus plasmid pUC-crtE (*E. limosum crtE*) (2), plus plasmid pUC-crtE (L. *mesenteroides crtE*) (3), and plus plasmid pUC-crtE (L. *plantarum crtE*) (4).

GGPP is synthesized from farnesyl diphosphate (FPP) by CrtE (GGPP synthase) [37]. C_40_-carotenoids (e.g., phytoene, lycopene, β-carotene, and zeaxanthin) can be subsequently synthesized. To evaluate the enzymatic activity of the three identified CrtE candidates (orthologues) from *E. limosum,* L. *mesenteroides*, and L. *plantarum*, we used *E. coli* transformed with plasmid pACCAR25ΔcrtEX, which includes *crtB, crtI, crtY*, and *crtZ*, as the host. Since this strain retains very low endogenous GGPP synthase activity, a small amount of zeaxanthin should be produced. When the empty pUC19 vector was introduced into *E. coli* containing pACCAR25ΔcrtEX, the cells exhibited a pale yellow color. Fig. 6 shows the result of the carotenoid production test by the *E. coli* transformants. A significant difference (*P* < 0.01) was present between the transformants with the empty pUC19 vector and those with pUC-crtE of L. *mesenteroides* or L. *plantarum*, though not a difference with that of *E. limosum.* The result thus suggests that the CrtE proteins from L. *mesenteroides* and L. *plantarum* exerted low GGPP synthase activity.

## 4 Discussion

Wieland et al. [40] initially demonstrated, through functional analysis with *E. coli*, that CrtM derived from *S. aureus* (family Staphylococcaceae, class Bacilli) converts FPP into 4,4′-diapophytoene, subsequently metabolized to 4,4′-diaponeurosporene with the *S. aureus* CrtN. Since then, numerous *crtM* and *crtN* (also known as *crtNa*) gene sequences have been identified in bacteria primarily belonging to the class Bacilli, as examples described in Table 2. Regarding lactic acid bacteria (family Lactobacillaceae, class Bacilli), L. *plantarum* and *Enterococcus gilvus* were reported to retain *crtM* and *crtN* genes, functioning similarly to those of *S. aureus* [25,26,43,44]. In this study, we extend this category by adding the *crtM* and *crtN* genes of L. *mesenteroides* subsp. *mesenteroides* [family Lactobacillaceae (previously Leuconostocaceae)] However, limited research has been conducted on the structure and function of carotenoid biosynthesis genes in human gut bacteria (excluding lactic acid bacteria). Here, we discovered carotenoid biosynthesis genes, *crtM* and *crtN*, in a human gut bacterium *E. limosum* (family Eubacteriaceae, class Clostridia). These isolated carotenogenic genes were functionally identified using the *E. coli* functional expression system, followed by chemical analysis of the generated natural products.

Turpin et al. [43] identified the GGPP synthase gene (*crtE*), alongside the carotenoid biosynthesis genes *crtM* and *crtN*, in the genomes of several bacteria belonging to the Lactobacillaceae family. However, it has not been examined whether these *crtE* genes function or not. We also found *crtE* orthologs in *E. limosum* in addition to L. *mesenteroides* and L. *plantarum.* Functional analysis of these genes revealed no or very weak GGPP synthase activities. This result suggests that the *crtE* orthologues in gut and lactic acid bacteria are not involved in carotenoid biosynthesis. This is likely one of the reasons why Firmicutes is unable to produce C_40_-carotenoids, even though *crtM* has the potential to synthesize a C_40_-carotenoid [45], similar to L. *plantarum crtM*.

**Fig. 7:**
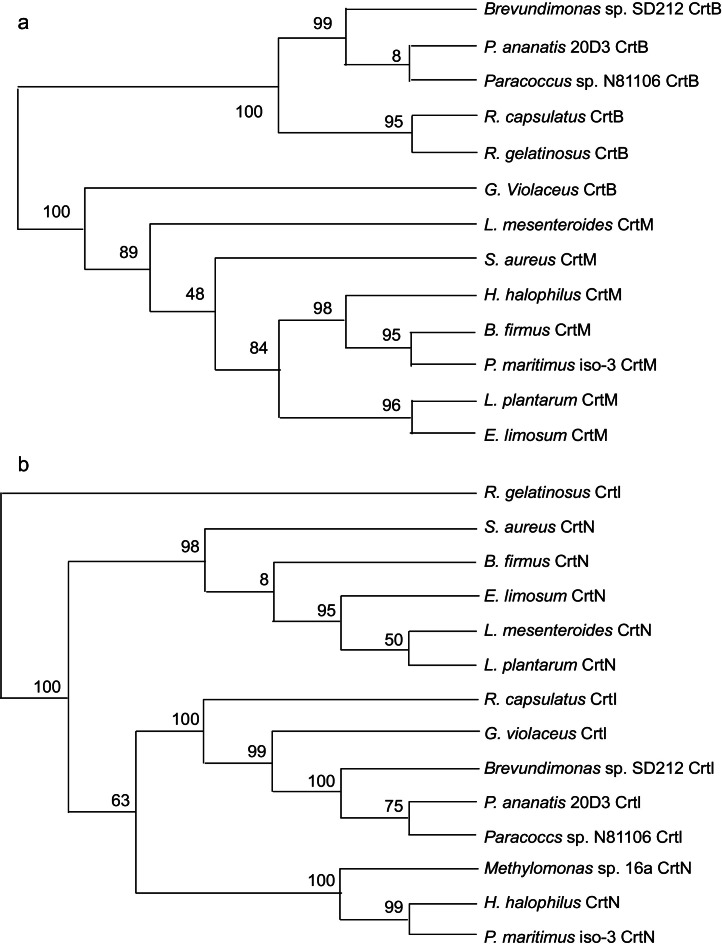
Phylogenetic trees of CrtM and CrtB (**a**) and CrtN and CrtI (**b**). Accession numbers of individual proteins are described in Table 2.

Evolutionary evidence suggests the emergence of a pathway for C_30_ carotenoids originating from the squalene synthetic route [27]. In Fig. 7a, a phylogenetic tree of representative 4,4′-diapophytoene synthase (CrtM) and phytoene synthase (CrtB) proteins (Table 2a) reveals distinct clades for CrtM and CrtB proteins. Notably, the CrtB protein of the primitive cyanobacterium *Gloeobacter violaceus* is included in the CrtM clade, indicating an early branching point. Our study also uncovered that the L. *plantalum* CrtM possesses both CrtM and CrtB activities, suggesting an evolutionary transition from CrtM to CrtB, a feasibility supported by directed evolution experiments [45].

**Table 2a:** Examples of the *crtM* and *crtB* gene products (proteins) and their accession numbers.

Organism	Gene	Accession no.
*Eubacterium limosum*	*crtM*	ARD66429.1
*Leuconostoc mesenteroides* subsp. *mesenteroides*	*crtM*	ABJ62147.1
*Lactobacillus* (*Lactiplantibacillus*) *plantarum*	*crtM*	AJO75313.1
*Staphylococcus aureus*	*crtM*	AMQ79551.1
*Halobacillus halophilus* DSM2266	*crtM*	ACM07426.1
*Planococcus maritimus* strain iso3	*crtM*	LC620265
*Bacillus* (*Cytobacillus*) *firmus*	*crtM*	EWG10945.1
*Gloeobacter violaceus* PCC7421	*crtB*	BAC89685.1
*Rhodobacter capsulatus* SB1003	*crtB*	ADE84445.1
*Rubrivivax gelatinosus*	*crtB*	AAB87738.2
*Pantoea ananatis* 20D3	*crtB*	BAA14128.2
*Paracoccus* sp. strain N81106	*crtB*	BAA09595.2
*Brevundimonas* sp. strain SD212	*crtB*	BAD99409.1

In Fig. 7b, a phylogenetic tree of representative 4,4′-diapophytoene desaturase (CrtN) and phytoene desaturase (CrtI) proteins (Table 2b) displays distinct clades for CrtI and two clades for CrtN. The first branch point in the CrtI clade features CrtI of the purple non-sulfur photosynthetic bacterium *Rubrivivax gelatinosus*, capable of producing both neurosporene and lycopene [46]. In the CrtI clade, CrtI of a purple non-sulfur photosynthetic bacterium *Rhodobacter capsulatus*, which catalyses neurosporene production [36,47]. The other CrtI proteins in this clade exhibit typical lycopene synthase activity [36,37]. Regarding CrtN in Fig. 7b (Table 2b), enzymes from *Bacillus firmus* (renamed *Cytobacillus firmus*) and *Methylomonas* sp. strain 16a can synthesize 4,4′-diapolycopene via 4,4′-diaponeurosporene [48,49], while the remaining CrtN enzymes synthesize 4,4′-diaponeurosporene. The distinction in reaction specificity is challenging to discern from primary structure alone, unlike the case of CrtI.

**Table 2b:** Examples of the *crtN* and *crtI* gene products (proteins) and their accession numbers.

Organism	Gene	Accession no.
*Eubacterium limosum*	*crtN* (*crtNa*)	ARD66430.1
*Leuconostoc mesenteroides* subsp. *mesenteroides*	*crtN* (*crtNa*)	ABJ62146.1
*Lactobacillus* (*Lactiplantibacillus*) *plantarum*	*crtN* (*crtNa*)	AJO75312.1
*Staphylococcus aureus*	*crtN* (*crtNa*)	WP_057520765.1
*Halobacillus halophilus* DSM2266	*crtN* (*crtNa*)	ACM07424.1
*Methylomonas* sp. strain 16a	*crtN* (*crtNa*)	AAX46183.1
*Planococcus maritimus* strain iso3	*crtN (crtNa)*	LC620265
*Bacillus* (*Cytobacillus*) *firmus*	*crtN* (*crtNa*)	AGX02541.1
*Gloeobacter violaceus* PCC7421	*crtI*	BAC88808
*Rhodobacter capsulatus* SB1003	*crtI*	ADE84444.1
*Rubrivivax gelatinosus*	*crtI*	WP_043810772.1
*Pantoea ananatis* 20D3	*crtI*	BAA14127.1
*Paracoccus* sp. strain N81106	*crtI*	BAA09594.2
*Brevundimonas* sp. strain SD212	*crtI*	BAD99408.1

In our investigation, pure-cultured human intestinal bacteria, including gut and probiotic strains, did not exhibit the presence of carotenoids under strictly anaerobic conditions mimicking the human intestinal environment. However, when L. *mesenteroides* and L. *plantarum* were cultured under semi-aerobic conditions, distinct from the anaerobic environment of the human gut, small amounts of 4,4′-diaponeurosporene were detected. This suggests that C_30_ carotenoids may not be produced within the human gut microbiota, but lactic acid bacteria harboring *crtM* and *crtN* genes, such as L. *mesenteroides* and L. *plantarum*, could produce 4,4′-diaponeurosporene when exposed to aerobic environments outside the gut.

Regarding C_40_ carotenoids, carotenoids, our findings and reasoning suggest that the human intestinal microbiota is unlikely to biosynthesize these pigments. If the intestinal microbiota possessed the capability to produce C_40_ carotenoids at levels comparable to vitamin K, they would serve as a significant source supplying C_40_ carotenoids like β-carotene or β-cryptoxanthin to humans. In such a scenario, Vitamin A deficiency symptoms would be unlikely to occur. Moreover, the examined bacteria in this study lacked gene sequences for C_40_ carotenoid biosynthesis, such as *crtY* and *crtZ*, and the *crtE* gene coding for functional GGPP synthase was also absent in the examined bacteria.

*E. limosum* is an obligate anaerobic bacterium thriving on carbon monoxide (CO) as its exclusive carbon/energy source, yielding acetate, butylate, and carbon dioxide (CO_2_) as major products [50,51]. Notably, *E. limosum* exhibits *O*-demethylation capabilities for certain methoxylated low-molecular weight compounds like isoflavonoids [52]. Given these unique characteristics, *E. limosum* is deemed physiologically significant among human gut bacteria. In our investigation, it was surprising to discover that *E. limosum* harbors functional *crtM* and *crtN* genes, despite not producing carotenoids in pure culture environments with GAM medium under strictly anaerobic conditions. This unexpected result suggests that these obligate anaerobes retain carotenoid biosynthesis genes, potentially with no immediate self-utility, which become active only in aerobic conditions. The possibility arises that this microbe may be transported to aerobic environments through bowel movements or undergo mutations that chance upon adaptation to aerobic circumstances. In such cases, the biosynthesized carotenoids could confer survival advantages. This unique aspect of carotenoid biosynthesis, where a gene is arranged in advance before its immediate utility, may provide insights into the evolutionary direction of biosynthesis. In the future, synthetic biology could potentially engineer the metabolic system of microbes like *E. limosum* to adapt to aerobic conditions or express carotenogenic genes functionally even in the absence of oxygen molecules.

## Data Availability Statement

Data used in this study are available on request to the corresponding authors. Main plasmids are available from Riken BRC (https://web.brc.riken.jp/en/).

## CRediT authorship contribution statement

**Wataru Matsumoto:** Investigation. **Miho Takemura:** Writing – original draft, Investigation. **Haruka Nanaura:** Investigation. **Yuta Ami:** Investigation. **Takashi Maoka:** Investigation. **Kazutoshi Shindo:** Writing – original draft, Investigation. **Shin Kurihara:** Validation, Resources. **Norihiko Misawa:** Writing – review & editing, Writing – original draft, Supervision, Conceptualization.

## Declaration of Competing Interest

All the authors declare that they have no known competing financial interests or personal relationships that could have appeared to influence the work reported in this research article.

## Appendix Supplementary materials

Image, application 1
