# Supplementary material for: Carotenoid productivity in human intestinal bacteria Eubacterium limosum and Leuconostoc mesenteroides with functional analysis of their carotenoid biosynthesis genes
Source: Eng Microbiol. 2024 Mar 28;4(2):100147. doi: 10.1016/j.engmic.2024.100147 (PMC11611032; doi:10.1016/j.engmic.2024.100147)
Supplement: Supplementary file 1 [file mmc1.docx]

**Supplementary Materials**

**Carotenoid productivity in human intestinal bacteria *Eubacterium limosum* and *Leuconostoc mesenteroides* with functional analysis of their carotenoid biosynthesis genes**

Wataru Matsumoto, Miho Takemura, Haruka Nanaura, Yuta Ami, Takashi Maoka, Kazutoshi Shindo, Shin Kurihara, and Norihiko Misawa

**Table S1.** Primer sequences used in this study for isolation of carotenoid biosynthesis gene homologues from human intestinal bacteria

*Bv, *B. vulgatus*; Pd, *P. distasonis*; Bo, *B. ovatus*; Bd, *B. dorei*; Cp, *C. perfringens*; El, *E. limosum*; Lp, *L. plantarum*; Lm, *L. mesenteroides*. **underline, restriction enzyme site.

**Table S2.** 16S rDNA sequences read from cultured bacteria

**Figure S1.** Colonies after the strictly anaerobic culture in the 96-well plates without agitation at 37°C for 24 h

**Figure S2.** HPLC chromatograms with UV-visible spectra of extracts from *E. coli* cells that retained plasmid pAC-HIM (*L. plantarum crtM*) plus the empty vector pUC18 (**a**), plus plasmid pUC-crtN (*B. dorei crtN*) (**b**), plus plasmid pUC-crtN (*B. ovatus crtN*) (**c**), plus plasmid pUC-crtN (*B. vulgatus crtN*) (**d**), plus plasmid pUC-crtN (*C. perfringens crtN*) (**e**), and plus plasmid pUC-crtN (*P. distasonis crtN*) (**f**). **1**, 4,4’-diapophytoene.
